# Long Non-Coding RNAs and Micro RNAs in Chronic Kidney Disease: Recent Advances and Future Directions—A 5-Year Systematic Review

**DOI:** 10.3390/life16040579

**Published:** 2026-04-01

**Authors:** Kanellos Skourtsidis, Despoina Ioannou, Georgios Kiosis, Konstantinos Stergiou, Maria Nefeli Georgaki, Theodora Papamitsou, Sofia Karachrysafi

**Affiliations:** 1 Research Team “Histologistas”, Interinstitutional Postgraduate Program “Health and Environmental Factors”, Medical School, Faculty of Health Sciences, Aristotle University of Thessaloniki, 54124 Thessaloniki, Greece; dioana@auth.gr (D.I.); gkiosis@auth.gr (G.K.); konstantinos.d.stergiou@outlook.com (K.S.); nefgeor@gmail.com (M.N.G.); thpapami@auth.gr (T.P.); sofia_karachrysafi@outlook.com (S.K.); 2 Laboratory of Histology-Embryology, Medical School, Faculty of Health Sciences, Aristotle University of Thessaloniki, 54124 Thessaloniki, Greece; 3 Environmental Engineering Laboratory (EnvE-Lab), Department of Chemical Engineering, Aristotle University of Thessaloniki, 54124 Thessaloniki, Greece

**Keywords:** Chronic Kidney Disease (CKD), non-coding RNAs (ncRNAs), microRNAs (miRNAs), long non-coding RNAs (lncRNAs), AKI-to-CKD transition, biomarkers, therapeutic targets

## Abstract

Introduction: Chronic Kidney Disease (CKD) is a leading public health problem worldwide, with limited therapeutic options to halt its progression. Recent evidence implicates non-coding RNAs (ncRNAs), specifically long non-coding RNAs (lncRNAs) and microRNAs (miRNAs), as critical regulators in renal pathophysiology and the transition from Acute Kidney Injury (AKI) to CKD. This review aims to synthesize recent findings regarding the role of ncRNAs in CKD pathogenesis, emphasizing their potential as diagnostic biomarkers and therapeutic targets. Methods: A systematic search was conducted in the PubMed/MEDLINE and Scopus databases for original research articles published over the last five years. Studies were selected based on specific eligibility criteria focusing on the correlation of ncRNAs with the development, diagnosis, and therapy of CKD. A total of 14 studies were included in the final review. Results: This review identified a dual landscape of ncRNAs function. Several lncRNAs, including H19, MALAT1, NEAT1_2, and LINC00963, were found to act as pathogenic drivers, promoting inflammation, apoptosis, and fibrosis through pathways such as TGF-β/Smad and NF-κB. Specifically, MALAT1 and NEAT1_2 are pivotal in driving the AKI-to-CKD transition. Conversely, specific miRNAs, such as miR-204, miR-26a, miR-451, miR-101, and miR-486-5p, exhibited protective effects by attenuating oxidative stress, preserving endothelial function, and inhibiting epithelial–mesenchymal transition (EMT). Dysregulation of these molecules was also observed in systemic conditions affecting the kidney, such as congestive heart failure and β-thalassemia. Conclusions: ncRNAs are central players in the molecular mechanisms underlying renal injury and maladaptive repair. The identified lncRNAs and miRNAs offer promising avenues for non-invasive diagnosis and the development of novel targeted therapies to prevent fibrosis and slow the progression of CKD.

## 1. Introduction

### 1.1. Chronic Kidney Disease (CKD)

Chronic Kidney Disease (CKD) is defined as a persistent reduction in renal function or structural kidney damage lasting at least three months, typically reflected by a GFR < 60 mL/min/1.73 m^2^ or markers such as proteinuria or hematuria [1,2]. CKD affects over 10% of the global adult population, with more than 800 million individuals impacted worldwide [3,4,5]. Beyond its high prevalence, CKD substantially increases cardiovascular morbidity and mortality, particularly in advanced stages, where complications such as coronary artery disease and heart failure are common [6,7,8]. Despite its clinical significance, early detection remains challenging, underscoring the need for novel molecular biomarkers.

### 1.2. Risk Factors for Chronic Kidney Disease

CKD development is influenced by non-modifiable factors such as age-related nephron loss and genetic predisposition, including APOL1 risk alleles [9,10]. Major modifiable contributors include hypertension, diabetes mellitus, obesity, and proteinuria, all of which accelerate nephron injury and disease progression [9,11,12,13,14,15,16,17,18]. Additional risks arise from dyslipidemia, smoking, high sodium intake, environmental toxins, and chronic exposure to nephrotoxic drugs such as NSAIDs [19,20,21,22,23,24,25]. These diverse factors highlight the multifactorial nature of CKD and the need for molecular tools capable of detecting early pathological changes.

### 1.3. Acute Kidney Injury to Chronic Kidney Disease Transition

Acute Kidney Injury (AKI) is now recognized as a major driver of CKD, as even clinically recovered episodes may leave behind maladaptive repair processes such as tubular atrophy, interstitial fibrosis, and capillary rarefaction [2,26]. The risk of CKD increases with the severity and recurrence of AKI and is further amplified by comorbidities like diabetes and hypertension [27,28]. At the molecular level, mitochondrial dysfunction, persistent inflammation, ferroptosis, and dysregulation of non-coding RNAs have been implicated in the AKI-to-CKD transition [29,30,31,32], positioning ncRNAs as key regulators of long-term renal outcomes.

### 1.4. Non-Coding RNAs and Their Role in CKD

The human genome is pervasively transcribed, yet only a small fraction encodes proteins. The majority of transcripts are classified as non-coding RNAs (ncRNAs), which exert regulatory functions at transcriptional, post-transcriptional, and epigenetic levels [33]. NcRNAs are broadly categorized into small non-coding RNAs, such as microRNAs (miRNAs), and long non-coding RNAs (lncRNAs), as well as circular RNAs (circRNAs) that form covalently closed loops [34,35,36].

NcRNAs are increasingly recognized as central players in renal pathophysiology. MicroRNAs regulate gene expression by targeting messenger RNAs for degradation or translational repression, and their dysregulation has been implicated in renal fibrosis, inflammation, and podocyte injury [37,38,39]. Long non-coding RNAs act through diverse mechanisms, including chromatin remodeling and transcriptional interference, and act as molecular sponges for miRNAs. Several lncRNAs, such as MALAT1 and NEAT1, have been linked to tubular epithelial cell injury and progression of CKD [40,,41]. Meanwhile, circular RNAs are emerging as important regulators of renal disease, with roles in oxidative stress and epithelial–mesenchymal transition (EMT), both of which contribute to tubulointerstitial fibrosis [42,43].

Beyond functional roles, ncRNAs hold promise as biomarkers and therapeutic targets. Their high stability in biological fluids, tissue specificity, and dynamic expression profiles make them attractive candidates for non-invasive biomarkers of CKD progression [44,45]. In addition, experimental modulation of ncRNAs using mimics, inhibitors, or CRISPR-based tools has shown potential in ameliorating renal fibrosis and inflammation in preclinical models, underscoring their translational relevance [46,47]. Thus, ncRNAs represent a rapidly evolving field in nephrology, offering novel insights into the molecular mechanisms that underlie the initiation and progression of CKD, and providing potential opportunities for earlier diagnosis and innovative therapeutic strategies.

Given the increasing global burden of chronic kidney disease (CKD), the limited efficacy of current therapies, and the emerging evidence that non-coding RNAs (ncRNAs) play crucial roles in renal inflammation, fibrosis, and maladaptive repair, there is a pressing need to synthesize recent insights in this field. This study aims to provide an updated overview of the role of ncRNAs, particularly microRNAs and long non-coding RNAs, in the pathogenesis and progression of CKD, emphasizing their potential as diagnostic biomarkers and therapeutic targets. By highlighting recent advances, we seek to bridge the gap between experimental discoveries and clinical translation, thereby contributing to the development of novel strategies for early detection and intervention in CKD.

## 2. Materials and Methods

### 2.1. Search Strategy and Eligibility Criteria

A systematic and structured literature search was performed on 7 April 2025, to identify relevant studies investigating the role of non-coding RNAs in chronic kidney disease (CKD). We searched two primary electronic databases, PubMed/MEDLINE and Scopus, for original research articles published between 2020 and 2025 in the English language. To ensure transparency and reproducibility, the following exact Boolean search strings were utilized: “chronic kidney injury” AND “lncRNA” and “chronic kidney injury” AND “miRNA”, as well as synonymous terms. These strings were designed to capture the intersection of renal injury and specific non-coding RNA subtypes.

### 2.2. Eligibility Criteria and Study Selection

Studies were selected based on the PICO (Population, Intervention, Comparison, Outcome) framework as detailed in Table 1. We included original research articles, prospective and retrospective cohort studies, and case reports available as open-access publications. The primary inclusion criterion focused on studies correlating ncRNAs with the development, diagnosis, and therapy of chronic kidney disease. We excluded publications not meeting the PICO framework and those published in languages other than English. No automation tools were used at any stage of the selection process; all screening of titles, abstracts, and full texts was performed manually by two independent reviewers to ensure data integrity.

### 2.3. Data Management

**Figure 1:**
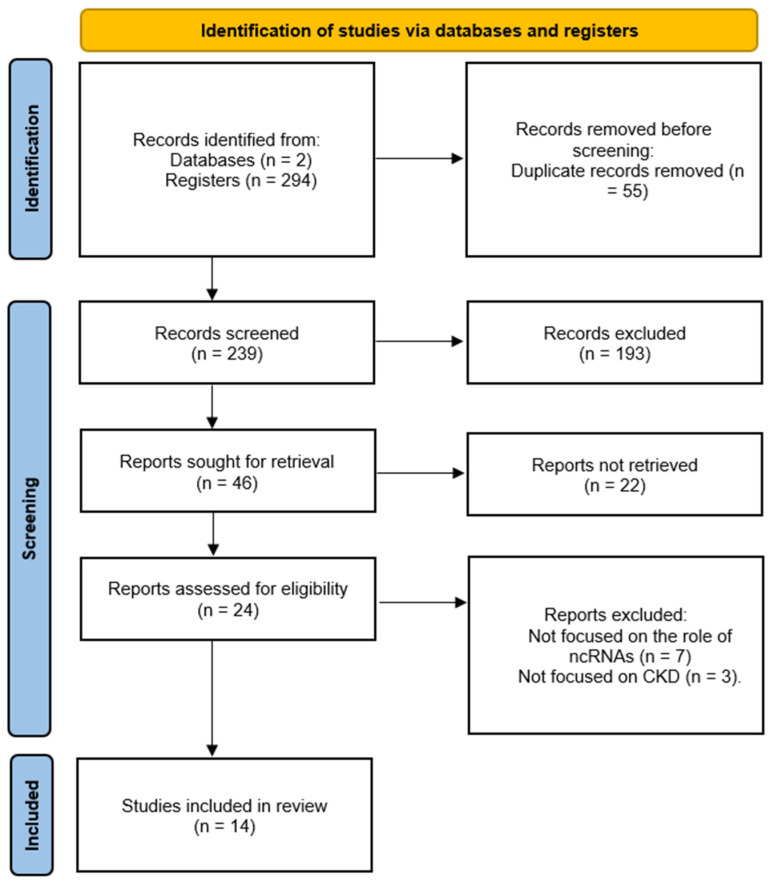
PRISMA Flow Diagram.

Regarding our data management, potentially eligible articles were exported into “Rayyan”, a web and mobile app for systematic reviews application for article screening (Rayyan Systems Inc., Cambridge, MA, USA, 2025). Via Rayyan, duplicates were removed. Two reviewers independently screened titles and abstracts, and subsequently, full texts, remaining blinded to each other’s decisions throughout the process. Conflicts were resolved with the involvement of a third investigator. The PRISMA flow diagram was constructed and presents the progress from initial studies to final results (Figure 1).

### 2.4. Risk of Bias

To ensure the methodological rigor of this review, the 14 selected studies underwent a rigorous quality assessment using the Joanna Briggs Institute (JBI) Critical Appraisal Tools. Given the diverse nature of the evidence—varying from human clinical cohorts to molecular laboratory experiments—the appraisal was tailored by matching each study to its specific JBI checklist according to its design. The distribution of the tools applied is summarized in Table 2 and Table 3.

Specifically, the JBI Checklist for Quasi-Experimental Studies was used for the 7 studies primarily focused on laboratory-based mechanistic insights and animal models, where interventions like gene silencing or induced ischemia were performed. Human observational data were evaluated using the Analytical Cross-Sectional tool for point-in-time comparisons and the Cohort tool for longitudinal outcome tracking. Finally, the matched comparison of patients in the study by Lu HY (2021) [50] was assessed via the Case-Control checklist. The detailed allocation of the JBI tools for each included study is presented in Table 3.

This systematic approach ensured that the strength of the evidence was verified across all experimental and clinical domains, supporting the validity of the conclusions regarding the roles of lncRNAs and miRNAs in CKD.

## 3. Results

### 3.1. Prisma Flow Diagram

The initial database search performed on 7 April 2025, yielded a total of 294 results across the two search categories. The search for “chronic kidney injury AND lncRNA” resulted in 57 hits from PubMed and 42 hits from Scopus. The search for “chronic kidney injury AND miRNA” yielded 195 hits from PubMed and 85 hits from Scopus. After the removal of 55 duplicates, 239 unique records were screened for eligibility.

During the first round of title and abstract screening, 193 articles were excluded as ineligible due to a lack of relevance to ncRNAs or CKD, the absence of original data such as editorials or non-systematic reviews, or restricted accessibility. Of the remaining 46 articles assessed in full-text, 24 were evaluated for final eligibility. Ultimately, 14 studies met all criteria and were included in the final review. The detailed selection process, including specific reasons for exclusion and the progress from initial identification to final inclusion, is illustrated in the PRISMA flow diagram in Figure 1. Protocol was not registered in PROSPERO. A full PRISMA check list can be found in Supplementary Material (File S1).

### 3.2. Risk of Bias

The methodological quality of the 14 included studies was robust, with all papers meeting the criteria for inclusion after critical appraisal. For the quasi-experimental studies (n = 7) (Table 4), which primarily utilized animal models and cell lines, there was a high level of internal validity; all studies clearly established a temporal cause-and-effect relationship (Q1) and utilized appropriate control groups (Q2) and reliable measurement techniques (Q7). While these studies did not employ multiple pre- and post-intervention measurements (Q5) due to the terminal nature of tissue analysis in molecular research, the consistency of the results across groups supported their inclusion. In the human observational domain, the analytical cross-sectional studies (n = 4) (Table 5) demonstrated clear inclusion criteria and standardized clinical definitions for CKD. While potential confounding factors were identified in most cases, some studies did not explicitly detail strategies to address these confounders (Q6), a common limitation in discovery-phase RNA profiling. Conversely, the cohort studies (Table 6) and the case–control study (Table 7) proved high methodological rigor, effectively identifying confounders and ensuring that participants were free of the primary outcome at the start of the observation period. Statistical analyses across all 14 studies were found to be appropriate for the data types presented. Consequently, the overall risk of bias was considered low to moderate, lending significant weight to the synthesized findings on the regulatory roles of lncRNAs and miRNAs in renal pathophysiology.

### 3.3. Main Results

#### 3.3.1. Long Noncoding RNAs (lncRNAs)

Long noncoding RNAs (lncRNAs) have emerged as essential regulatory molecules in the complex pathogenesis of Chronic Kidney Disease (CKD). These transcripts, while not translated into proteins, act as critical mediators of gene expression, often functioning as “molecular sponges” for microRNAs (miRNAs) or as structural scaffolds for signaling complexes. The following sections detail the involvement of specific lncRNAs, specifically H19, MALAT1, Neat1_2, PVT1, and LINC00963, in driving renal dysfunction, systemic inflammation, and the deleterious transition from AKI to CKD.

##### Human Observational and Translational Studies

Clinical investigations provide primary evidence for the utility of lncRNAs as both diagnostic biomarkers and systemic drivers of kidney pathology. These studies establish significant correlations between lncRNA expression levels and clinical parameters, such as the Glomerular Filtration Rate (GFR) and markers of mineral metabolism.

The study by Okuyan HM (2021) [49] utilized a cohort of 56 CKD patients and 20 healthy controls to investigate the clinical relevance of lncRNA H19. Findings revealed that H19 is significantly upregulated in the serum of CKD patients compared to healthy counterparts. Crucially, the expression levels of H19 demonstrated a strong negative correlation with the patients’ GFR, directly linking its elevation to impaired renal filtration capacity. Beyond its diagnostic value, Okuyan HM examined the relationship between H19 and the broader biochemical landscape of CKD. Elevated H19 expression was positively correlated with biomarkers of disordered mineral metabolism, specifically ferritin, phosphorus, and parathyroid hormone (PTH). Furthermore, H19 levels were closely tied to systemic inflammatory mediators, including Tumor Necrosis Factor-alpha (TNF-a) and Interleukin-6 (IL-6), as well as oxidative stress indicators such as osteocalcin, total antioxidant status (TAS), and total oxidant status (TOS). The study noted theHMGB1/TLR4/NF-ĸB signaling pathway that was promoted by the upregulation of lncRNA H19 in CKD patients. HMGB1 (High Mobility Group Box 1) is a nuclear protein that acts as a damage-associated molecular pattern (DAMP). In the extracellular space, HMGB1 binds to Toll-like receptor 4 (TLR4) on neighboring cells, initiating downstream inflammatory responses via NF-κB activation [62] These robust clinical correlations suggest that H19 serves as a central molecular node in the interplay between inflammation and oxidative stress that characterizes CKD progression and its associated systemic complications.

Lu HY (2021) [50] conducted a translational research study involving 129 AKI patients and 100 healthy controls. The results demonstrated that Metastasis Associated Lung Adenocarcinoma Transcript 1 (MALAT1) is markedly elevated in the clinical setting of acute kidney injury. The study identified a specific mechanistic axis where MALAT1 serves as a competitive endogenous RNA (ceRNA) or “decoy” for miR-204. By reducing the bioavailability of miR-204, MALAT1 facilitates the upregulation of Apolipoprotein L1 (APOL1), a gene intimately associated with glomerular and tubular injury. This molecular sequence leads to the activation of the NF-κB signaling pathway, prompting the massive production of pro-inflammatory cytokines such as IL-1β, IL-6, and TNFα. This clinical evidence underscores the role of MALAT1 in exacerbating the inflammatory storm and promoting programmed cell death (apoptosis) in the human kidney.

In a distinct clinical context, Chang Y (2021) [52] explored the role of Plasmacytoma Variant Translocation 1 (PVT1) in a study comprising 100 CHF patients, 50 CHF patients with concurrent CKD, and 50 healthy controls. Unlike other lncRNAs that are typically upregulated during disease, plasma PVT1 levels were found to be markedly downregulated in CHF patients. Notably, the lowest expression levels were observed in patients suffering from the “cardiorenal” phenotype (concurrent CHF and CKD). Longitudinal analysis revealed that patients with higher baseline PVT1 levels experienced a significantly lower incidence of developing CKD over a two-year follow-up period. Altered PVT1 expression may modulate inflammatory pathways, including NF-κB signaling, through interactions with miRNAs such as miR-124, as seen in vancomycin- or LPS-induced kidney injury [63]. This suggests that PVT1 exerts a protective effect on renal tissue, and its downregulation serves as a potent predictive marker for renal decline under cardiovascular stress.

The involvement of Neat1_2 (Nuclear Paraspeckle Assembly Transcript 1) was clinically validated by Ma T (2022) [51] in a cohort of 15 patients with AKI and 10 healthy controls. Researchers observed a significant upregulation of Neat1_2 in the kidney tissues of AKI patients. These clinical observations identified Neat1_2 as a molecular driver for the maladaptive repair process, facilitating the transition from acute injury to irreversible chronic disease.

##### Preclinical Evidence: Animal Models (In Vivo)

Animal models, particularly those involving ischemia-reperfusion injury (IRI) and high-phosphorus dietary interventions, provide deeper insights into the structural, fibrotic, and vascular damage induced by lncRNA dysregulation.

In a preclinical study using 60 Sprague Dawley (SD) rats, Qiang Liu (2022) [48] explored the impact of high-phosphorus diets on renal and vascular health. The study established that upregulated H19 promotes the H19/miR-138/TLR3 axis. In rats fed a high-phosphorus diet, H19 binds to miR-138, inhibiting its ability to suppress target mRNAs. This results in the over-expression of Toll-like receptor 3 (TLR3) [64]. The consequence of this cascade is the chronic activation of the NF-κB pathway, a pro-inflammatory and pro-calcification signaling sequence. This ultimately results in medial vascular calcification, a comorbid condition that severely deteriorates the long-term prognosis of CKD.

Ma T (2022) [51] utilized a mouse model subjected to ischemia-reperfusion (I/R) to study the dynamics of Neat1_2. The research revealed that under hypoxic conditions, the tumor suppressor p53 transcriptionally regulates and upregulates Neat1_2. This lncRNA then facilitates tubulointerstitial inflammation and fibrosis by acting as a molecular sponge for miR-129-5p. Experimental knockdown of Neat1_2 in these models preserved tubular integrity and significantly reduced the expression of extracellular matrix (ECM) components, highlighting its role as a therapeutic target to halt the progression from AKI to CKD.

Xie LB (2020) [53] investigated the LINC00963/miR-128-3p/JAK2/STAT1 axis in SD rats. Following renal I/R injury, LINC00963 expression was significantly elevated. The study found that silencing LINC00963 improved renal histology and reduced the phosphorylation of Janus Kinase 2 (JAK2) and Signal Transducer and Activator of Transcription 1 (STAT1). These are key components of the signaling pathway driving maladaptive epithelial injury and subsequent cellular apoptosis, fundamental factors of AKI to CKD progression. These findings support a model in which the LINC00963/miR-128-3p/JAK2/STAT1 axis drives maladaptive epithelial injury and apoptosis in AKI [65]. Targeting LINC00963,either by restoring miR-128-3p function or directly inhibiting LINC00963, holds therapeutic potential to attenuate tubular damage and prevent the progression from AKI to CKD.

##### Mechanistic Insights: Cell Line Studies (In Vitro)

In vitro experiments, particularly those utilizing human HK-2 cells (immortalized proximal tubule epithelial cells), have pinpointed the exact signaling cascades and molecular interactions governed by these lncRNAs.

The work of Ma T (2022) [51] and Xie LB (2020) [53] utilized HK-2 cells to model hypoxia-induced damage. In TECs, Neat1_2 prevents miR-129-5p from downregulating pro-apoptotic factors. This results in the elevation in FADD (Fas-associated protein with death domain), caspase-8, and caspase-3, thereby facilitating programmed cell death [66,67]. Moreover, elevated LINC00963 was found to target miR-128-3p directly. This interaction promotes G1 phase arrest and apoptosis in HK-2 cells via the JAK2/STAT1 pathway. Silencing LINC00963 was shown to mitigate these apoptotic effects, suggesting a potential therapeutic pathway for reducing tubular attrition.

Lu HY (2021) [50] used cellular models to demonstrate that under hypoxic conditions, MALAT1 expression is significantly induced while miR-204 levels are suppressed. MALAT1 serves as a decoy, preventing miR-204 from targeting APOL1. The resulting increase in APOL1 expression triggers NF-κB activation, leading to the secretion of pro-inflammatory cytokines and the promotion of apoptosis in renal cells.

The mechanistic study by Puri B (2025) [46] utilized laboratory models to demonstrate how MALAT1 drives the transition to renal fibrosis. Upregulated MALAT1 in injured renal cells promotes the phosphorylation and subsequent nuclear translocation of Smad2/3, the primary downstream effectors of the TGF-β pathway. This leads to the increased transcription of hallmark fibrotic markers, including collagen I, fibronectin, α-SMA (Alpha-Smooth Muscle Actin) and Vimentin [68,69]. Furthermore, the study highlighted that MALAT1 suppresses the inhibitory Smad7, which normally acts as a natural “brake” on TGF-β signaling. By removing this internal inhibition, MALAT1 sustains a chronic pro-fibrotic response. Conversely, experimental MALAT1 knockout (KO) was shown to attenuate this fibrosis, reducing the levels of p-Smad2, p-Smad2/3, and Smad4, thereby effectively preventing the AKI-to-CKD transition. This study provided evidence of a mitochondrial protective effect associated with the loss of MALAT1. In TGF-β induced models of the AKI-to-CKD transition, the absence of MALAT1 protected cells from the metabolic and structural shifts that typically precede chronic fibrosis. By inhibiting the MALAT1/Smad2/3 pathway, cells displayed significantly reduced levels of fibrotic markers, emphasizing the potential for MALAT1-targeted strategies to mitigate the transition from acute injury to chronic scarring.

The most important findings regarding lncRNAs are presented at Table 8.

#### 3.3.2. microRNAs (miRNAs)

MicroRNAs (miRNAs) have emerged as central post-transcriptional regulators in the pathophysiology of renal diseases. These small, non-coding RNA molecules exert their influence by binding to the 3′-untranslated regions (UTRs) of target mRNAs, leading to translational repression or mRNA degradation. In the context of the kidney, miRNAs function as “molecular switches” that govern critical processes such as inflammation, epithelial-to-mesenchymal transition (EMT), oxidative stress, and fibroblast activation. The following section categorizes recent evidence into clinical observations, animal models, and cellular mechanisms to highlight the diagnostic and therapeutic potential of these molecules.

##### Clinical and Human Studies

Xiaoyun G (2025) recently provided high-level clinical evidence regarding the significance of miR-204 in a large-scale observational study involving 126 patients with CKD and 126 healthy controls [54]. The study revealed that serum levels of miR-204 were significantly lower in the CKD cohort compared to healthy individuals. This reduction was not merely a byproduct of disease but was intricately linked to the severity of the pathology; higher serum levels of miR-204 were strongly correlated with better renal function, a reduced degree of interstitial fibrosis, and less tubular injury. Furthermore, miR-204 expression showed a significant negative association with inflammation scores. Patients with the lowest miR-204 levels exhibited the highest concentrations of pro-inflammatory cytokines, suggesting that a loss of miR-204-mediated regulation is a hallmark of CKD progression. Mechanistically, miR-204 has been shown to impair the MUC4-dependent activation of the ERK signaling pathway [70]. By inhibiting this pathway, miR-204 reduces oxidative stress and prevents the injury of tubular epithelial cells. Additionally, miR-204 protects podocytes from high glucose-induced apoptosis by downregulating Bdkrb2, further demonstrating its role in preserving the glomerular filtration barrier [71]. The protection afforded by miR-204 is closely linked to long non-coding RNA (lncRNA) networks. Under hypoxic conditions, the lncRNA MALAT1 is elevated, which acts as a “sponge” to suppress miR-204 availability [50].

In the realm of AKI and its long-term outcomes, especially CKD, Newbury LJ (2021) conducted a translational profiling study comparing urinary miRNA expression between AKI patients and healthy controls [55]. This study identified a distinct “miRNA signature” of injury: AKI patients showed marked increases in urinary miR-21, miR-126, and miR-141, while levels of miR-192 and miR-204 were significantly diminished. Of clinical importance, the elevation in urinary miR-141, when paired with the decrease in miR-192, served as a robust predictor of poor renal recovery at the 90-day mark. This underscores the potential for urinary miRNA profiling to serve as a “liquid biopsy” to identify patients at high risk for the AKI-to-CKD transition.

Specialized patient populations also demonstrate unique miRNA profiles. Ahmed HM (2022) focused on children with β-thalassemia major (β-TM), a group at high risk for CKD due to iron overload from frequent transfusions and the nephrotoxic potential of long-term chelation therapy [57,72]. While β-TM patients generally had higher plasma miR-451 levels than healthy children, those within the β-TM cohort who actually developed CKD exhibited significantly lower miR-451 expression compared to those without renal impairment [73]. Plasma miR-451 levels correlated positively with eGFR and reticulocyte counts, identifying it as an independent predictor of CKD onset in this vulnerable population. The mechanism behind miR-451 involves the YWHAZ gene, which encodes the 14-3-3 zeta protein [74]. Under normal conditions, miR-451 targets YWHAZ; however, when miR-451 is reduced, YWHAZ levels rise. High levels of 14-3-3 zeta sequester the transcription factor FoxO3 in the cytoplasm, preventing it from entering the nucleus to trigger the transcription of antioxidant enzymes like Catalase (Cat) and Glutathione Peroxidase-1 (GPx1). This impairment leads to runaway oxidative stress and tubular injury.

Similarly, Ji J (2022) addressed the critical issue of sepsis-induced AKI in pediatric patients, which carries high morbidity and mortality [58,75]. The study highlighted miR-320-3p as a promising prognostic biomarker [58]. Serum levels of miR-320-3p were significantly elevated in children with sepsis-AKI, particularly in those who did not survive. This miRNA’s expression correlated strongly with established markers of injury, including Neutrophil Gelatinase-Associated Lipocalin (NGAL), Kidney Injury Molecule-1 (KIM-1), and APACHE II scores. When used in combination with these traditional markers, miR-320-3p significantly enhanced the predictive accuracy for clinical outcomes and especially AKI to CKD transition, suggesting its utility in acute clinical settings.

##### Animal and In Vivo Models

A pivotal study by Ni W (2024) utilized miR-26a knockout (KO) mice to examine the miRNA’s role in Angiotensin II (Ang-II)-induced renal injury, a primary driver of hypertensive CKD [56]. In wild-type mice, Ang-II infusion led to a significant downregulation of miR-26a in the kidney. However, the miR-26a-KO mice exhibited far more severe outcomes, including aggravated albuminuria, elevated serum creatinine (SCr), and extensive renal fibrosis compared to controls. Most notably, the administration of exogenous miR-26a to these mice effectively attenuated proteinuria and suppressed both inflammation and fibrosis. This therapeutic “rescue” demonstrates that miR-26a is not just a marker of damage but a necessary protective factor against hypertensive renal remodeling. The protective effect of miR-26a is mediated through its direct targeting of LIMS1, a protein essential for stabilizing Integrin-Linked Kinase (ILK) [76]. When miR-26a is downregulated (as seen in CKD), the resulting activation of the LIMS1/ILK signaling pathway promotes extracellular matrix (ECM) deposition and the expression of pro-inflammatory cytokines such as IL-1β and IL-18 [77]. This cascade is a primary driver of fibroblast activation and renal scarring. These findings position miR-26a as both a biomarker and a therapeutic target in CKD, where restoring its expression could represent a novel strategy to suppress inflammation, mitigate fibrosis, and preserve kidney function.

Ischemia-reperfusion injury (IRI), a primary driver of AKI and a major factor in the transition to CKD is another major focus of in vivo research. Zhao JY (2020) demonstrated that miR-101 acts as a powerful anti-fibrotic agent in murine models of IRI-induced AKI [59]. Overexpression of miR-101 significantly reduced histological injury and suppressed markers of EMT, such as α-smooth muscle actin (α-SMA), vimentin, and various collagen subtypes (COL10A1, COL12A1), while successfully restoring the epithelial marker E-cadherin. This preservation of tubular structure is essential for preventing the maladaptive repair that leads to CKD. Krüppel-like factor 4 (KLF4) is as an upstream regulator of miR-101 [78]. Hypoxia or IRI decreases KLF4, which subsequently reduces miR-101 levels. Since miR-101 directly targets COL10A1, COL12A1, and TGFβ, its downregulation allows these pro-fibrotic genes to be overexpressed, leading to EMT and ECM accumulation [79]. Overexpressing KLF4 can rescue miR-101 levels, thereby suppressing α-SMA and vimentin and restoring E-cadherin expression [80].

Furthermore, Douvris A (2024) explored the potential of miR-486-5p as an early intervention in rat models of ischemic AKI [60]. Administration of miR-486-5p at the onset of reperfusion significantly reduced the rise in plasma creatinine and Blood Urea Nitrogen (BUN). The treatment also preserved renal cortical and medullary blood flow, preventing the “no-reflow” phenomenon and protecting the microvascular integrity by maintaining VEGFR2 expression and peritubular capillary density. These findings suggest that miR-486-5p could be a vital therapeutic candidate for preventing irreversible chronic injury during the acute phase of renal trauma. miR-486-5p exerts its renoprotective effects by targeting PTEN [81]. By modulating PTEN, this miRNA controls endothelial activation and inhibits the upregulation of ICAM-1 and TNF-α. This reduces leukocyte adhesion and migration, preventing the chronic inflammation that typically characterizes the transition from AKI to CKD [82].

##### Cell Line Studies (In Vitro)

At the cellular level, the specific pathways regulated by these miRNAs reveal how they control the fate of renal cells. miR-141, plays a dual role in renal health. In healthy kidneys, the miR-200 family maintains epithelial integrity by suppressing the transcription factors ZEB1 and ZEB2, which in turn preserves E-cadherin expression [83,84]. However, under oxidative stress, miR-141 is markedly upregulated in PTECs, where it targets Protein Tyrosine Phosphatase Receptor type G (PTPRG), as presented in Newbury LJ 2021 study [55]. Prolonged expression of miR-141, along with miR-21 and miR-126, leads to sustained EGFR activation and fibroblast activation [85]. This chronic signaling triggers the secretion of profibrotic cytokines like TGF-β, turning a repair mechanism into a driver of fibrosis [86]. The most important findings regarding miRNAs are presented at Table 9.

## 4. Discussion

### 4.1. Summary of Major Findings

Our study highlights the complex but critical role of non-coding RNAs in the pathophysiology of chronic kidney disease and emphasizes the progression from AKI to CKD. We analyzed several long non-coding RNAs including H19, MALAT1, NEAT1_2, PVT1, and LINC00963, as well as microRNAs such as miR-204, miR-141, miR-26a, miR-451, miR-320-3p, miR-101, and miR-486-5p. Collectively, these molecules influence renal inflammation, apoptosis, oxidative stress, fibrosis, and endothelial dysfunction, all of which are pivotal to kidney injury and maladaptive repair.

Our results suggest a dual landscape of ncRNAs: some lncRNAs (H19, MALAT1, NEAT1_2, LINC00963) act as pathogenic drivers, while several microRNAs (miR-204, miR-101, miR-486-5p) exert protective effects. Importantly, their dysregulation was not limited to local kidney injury but was also evident in systemic disease contexts, such as congestive heart failure and β-thalassemia, further underscoring their diagnostic and therapeutic relevance.

### 4.2. Integration with Previous Literature

Our findings are consistent with recent advances in ncRNAs research. For instance, lncRNA H19 was shown to negatively correlate with renal function while associating positively with inflammatory and metabolic markers, suggesting its role in accelerating CKD progression through inflammation and fibrosis pathways [87,88,89]. Similarly, MALAT1 has been widely implicated in AKI and CKD, due to the activation of pro-inflammatory and pro-fibrotic cascades [90,91]. In line with our observations, NEAT1_2 has been linked with AKI to CKD transition, facilitating tubular epithelial cell apoptosis and fibrosis [92,93,94]. All these mechanisms provide a strong basis for understanding how ncRNAs contribute to the maladaptive repair processes driving the initiation of CKD after AKI. For microRNAs, our study confirms protective roles for miR-204, miR-26a, miR-451, miR-101, and miR-486-5p. MiR-204 has previously been shown to inhibit oxidative stress damage in renal tubular cells and attenuate high-glucose and hypertension-induced podocyte apoptosis [95]. MiR-101 protects against renal fibrosis by inhibiting TGF-βR1-driven epithelial–mesenchymal transition (EMT) [80]. Likewise, miR-486-5p was demonstrated to preserve renal microvascular function after IRI, thereby preventing long-term fibrosis and systemic endothelial dysfunction [96].

The clinical relevance of these findings is strongly supported by the convergence of human and animal data. For example, while clinical studies demonstrate that elevated serum H19 correlates with decreased GFR and increased inflammatory markers in humans, preclinical models in Sprague Dawley rats provide the mechanistic explanation, showing that H19 actively drives vascular calcification via the TLR3/NF-κB axis. Similarly, the upregulation of MALAT1 observed in AKI patients is mirrored in animal models where its genetic knockout successfully prevents the AKI-to-CKD transition by inhibiting the TGF-β/Smad pro-fibrotic pathway.

### 4.3. Pathophysiological Insights

Not all ncRNAs participate in the same pathophysiological mechanisms. H19 [48] and MALAT1 [41] act as drivers of fibrosis and epithelial–mesenchymal transition (EMT) through activating TGF-β/Smad and NF-κB signaling. Moreover, miR-141 [55], miR-21 and miR-126 also promote the expression of profibrotic cytokines such as TGFβ1 and participate in EMT. In contrast with the previous, miR-26a [56], miR-486-5p [60] and mir-101 [59] attenuate fibrosis and EMT, and thus have a protective role. Regarding pro-apoptotic molecules, lncRNAs MALAT1 [41], NEAT1-2 [51], LINC00963 [53], and miR141 [55] promote cell death via caspase pathways or other signaling. Only miR-486-5p [60] was found to have a protective role against apoptosis, as it preserves endothelial function. Another important pathophysiological mechanism related to the pathogenesis of CKD or AKI-to-CKD transition is inflammation. LncRNA MALAT1 [50] and downregulation of miR-204 [54] seem to promote inflammatory reaction, whereas miR-451 [57] and miR 320-3p [58] have anti-inflammatory effects. The role of ncRNAs in CKD has also been investigated in the context of two systemic diseases, CHF and β-thalassemia major. In CHF-related CKD, PVT1 downregulation could serve as a diagnostic biomarker, despite its pro-injury role in sepsis and drug-induced AKI [52]. In β-thalassemia major, reduced miR-451 expression correlates with CKD and diminished antioxidant defenses, linking hematologic and renal pathology [57].

Comparison across different models reveals that the protective roles of certain miRNAs are consistent from rodents to humans. In human cohorts, low levels of miR-204 and miR-451 are robust predictors of renal dysfunction and oxidative stress. These observations are validated by ‘rescue’ experiments in in vivo models, such as those involving miR-26a knockout mice, where the administration of exogenous miR-26a directly attenuated albuminuria and suppressed renal fibrosis. This cross-species consistency underscores the potential of these molecules as reliable therapeutic targets.

### 4.4. Clinical Implications

The translational potential of the ncRNAs mentioned in this review is significant, offering non-invasive monitoring of renal structural health. PVT1, miR-204, miR-141, miR-192, and miR-320-3p already serve as robust biomarkers in serum or urine. While investigated in systemic conditions like sarcopenia, LEASO, colon or gallbladder cancer, and type 2 diabetes, they specifically reflect podocyte stress and tubular injury in the kidney [97,98,99,100,101]. In addition, for other ncRNAs, a potential therapeutic role is evident, either by targeting and knocking them out or by supplementing patients with them. For instance, inhibiting the MALAT1/Smad2/3, MALAT1/miR-204/APOL1/NF-κB, NEAT1_2, or LINC00963 pathways can retard CKD progression by halting tubulointerstitial fibrosis and epithelial cell apoptosis. These targets follow established research models in prostate, laryngeal, lung, and colorectal pathologies [102,103,104,105]. Conversely, supplementation with miR-26a, miR-451, miR-101, and miR-486-5p may slow the AKI-to-CKD transition by preserving peritubular capillary density and attenuating oxidative stress. Such strategies mirror therapeutic approaches used in gastric, lung, or hepatocellular cancers and pulmonary fibrosis [106,107,108,109].

### 4.5. Research Gaps and Future Directions

Despite the promising findings regarding the role of ncRNAs in renal pathophysiology, several critical research gaps remain that must be addressed to move the field toward clinical application. A primary requirement is the transition from observational associations to the establishment of definitive causality. While clinical investigations have established significant correlations between lncRNA expression levels and impaired renal filtration capacity, much of the evidence remains tied to descriptive data. Future research should utilize inducible, cell-specific knockout models to prove that these molecules are the primary drivers of structural and morphological alterations, such as interstitial fibrosis and tubular atrophy, rather than secondary markers of cellular stress. Furthermore, in order to ensure that urinary miRNA profiles can be reliably compared across clinical settings and serve as a “liquid biopsy”, research must focus on defining optimal reference genes and normalization techniques. Finally, future studies must achieve greater anatomical and compartmental specificity. The kidney is a complex organ, and understanding the “crosstalk” between different cell types is crucial. Integrating ncRNA data with other “omics” platforms, such as proteomics and metabolomics, will further clarify how the dysregulation of a single molecule alters the entire structural and metabolic landscape of the nephron.

### 4.6. Limitations

Despite strong preclinical evidence, several limitations must be acknowledged. Firstly, some studies were conducted in animal models or cultured cells, raising concerns about translatability to humans. Secondly, only English written articles available in Medline/PubMed and Scopus databases were retrieved; so, articles that are indexed exclusively in other databases are not represented herein. Thirdly, this is a systematic review; so, the results were not analyzed quantitatively.

## 5. Conclusions

In conclusion, this review clarifies the role of non-coding RNAs as functional mediators in the AKI-to-CKD transition. The synthesis of the findings reveals a consistent molecular dichotomy: specific lncRNAs (H19, MALAT1, NEAT1_2, and LINC00963) function as pathogenic participants that contribute to renal decline through the activation of pro-fibrotic and pro-inflammatory signaling pathways such as TGF-β/Smad and NF-κB in the renal tubulointerstitial compartment and tubular epithelial cells. Conversely, a signature of protective miRNAs, most notably miR-204, miR-26a, miR-101, and miR-486-5p, acts to preserve microvascular integrity by attenuating oxidative stress and inhibiting epithelial–mesenchymal transition. By bridging findings from human clinical cohorts with mechanistic animal models, this study identifies these ncRNA networks as robust candidates for early non-invasive diagnosis and targeted therapeutic intervention to halt the progression of CKD.

## Figures and Tables

**Table 1:** PICO table outlining Population (P), Intervention (I), Comparator (C), and Outcome (O).

PICO	
Population (P)	Adults with chronic kidney disease (CKD), including patients at risk due to systematic conditions, or history of acute kidney injury (AKI).
Intervention (I)	Role of non-coding RNAs in kidney injury, and progression of CKD.
Comparator (C)	Standard clinical biomarkers (e.g., creatinine, eGFR, albuminuria) or absence of ncRNAs evaluation.
Outcome (O)	Early diagnosis, prediction of CKD progression, identification of therapeutic targets, and potential improvement in disease management.

**Table 2:** Distribution of JBI Quality Assessment Tools.

JIB Quality Assessment Tool	JBI Checklist for Quasi-Experimental Studies	JBI Checklist for Analytical Cross-Sectional Studies	JBI Checklist for Cohort Studies	JBI Checklist for Case-Control Studies
Number of studies applied	7	4	2	1

**Table 3:** Analytical Application of JBI Tools per Study.

#	Study	JIB Quality Assessment Tool
1	Qiang Liu 2022 [48]	JBI Checklist for Quasi-Experimental Studies
2	Okuyan HM 2021 [49]	JBI Checklist for Analytical Cross-Sectional Studies
3	Puri B 2025 [46]	JBI Checklist for Quasi-Experimental Studies
4	Lu HY 2021 [50]	JBI Checklist for Case-Control Studies
5	Ma T 2022 [51]	JBI Checklist for Quasi-Experimental Studies
6	Chang Y 2021 [52]	JBI Checklist for Cohort Studies
7	Xie LB 2020 [53]	JBI Checklist for Quasi-Experimental Studies
8	Xiaoyun G 2025 [54]	JBI Checklist for Analytical Cross-Sectional Studies
9	Newbury LJ 2021 [55]	JBI Checklist for Analytical Cross-Sectional Studies
10	Ni W 2024 [56]	JBI Checklist for Quasi-Experimental Studies
11	Ahmed HM 2022 [57]	JBI Checklist for Analytical Cross-Sectional Studies
12	Ji J 2022 [58]	JBI Checklist for Cohort Studies
13	Zhao JY 2020 [59]	JBI Checklist for Quasi-Experimental Studies
14	Douvris A 2024 [60]	JBI Checklist for Quasi-Experimental Studies

**Table 4:** JBI Checklist for Quasi-Experimental Studies [61].

Domain Questions (Q)	Qiang Liu 2022 [48]	Puri B 2025 [46]	Ma T 2022 [51]	Xie LB 2020 [53]	Ni W 2024 [56]	Zhao JY 2020 [59]	Douvris A 2024 [60]
Q1: Is it clear in the study what is the “cause” and what is the “effect” (i.e., there is no confusion about which variable comes first)?	YES	YES	YES	YES	YES	YES	YES
Q2: Was there a control group?	YES	YES	YES	YES	YES	YES	YES
Q3: Were participants included in any comparisons similar?	YES	YES	YES	YES	YES	YES	YES
Q4: Were the participants included in any comparisons receiving similar treatment/care, other than the exposure or intervention of interest?	YES	YES	YES	YES	YES	YES	YES
Q5: Were there multiple measurements of the outcome, both pre and post the intervention/exposure?	NO	NO	NO	NO	NO	NO	NO
Q6: Were the outcomes of participants included in any comparisons measured in the same way?	YES	YES	YES	YES	YES	YES	YES
Q7: Were outcomes measured in a reliable way?	YES	YES	YES	YES	YES	YES	YES
Q8: Was follow-up complete and if not, were differences between groups in terms of their follow-up adequately described and analyzed?	YES	YES	YES	YES	YES	YES	YES
Q9: Was appropriate statistical analysis used?	YES	YES	YES	YES	YES	YES	YES
Overall appraisal	INCLUDE	INCLUDE	INCLUDE	INCLUDE	INCLUDE	INCLUDE	INCLUDE

**Table 5:** JBI Checklist for Analytical Cross-Sectional Studies.

Domain Questions (Q)	Okuyan HM 2021 [49]	Xiaoyun G 2025 [54]	Newbury LJ 2021 [55]	Ahmed HM 2022 [57]
Q1: Were the criteria for inclusion in the sample clearly defined?	YES	YES	YES	YES
Q2: Were the study subjects and the setting described in detail?	YES	YES	YES	YES
Q3: Was the exposure measured in a valid and reliable way?	YES	YES	YES	YES
Q4: Were objective, standard criteria used for measurement of the condition?	YES	YES	YES	YES
Q5: Were confounding factors identified?	YES	YES	UNCLEAR	YES
Q6: Were strategies to deal with confounding factors stated?	NO	YES	NO	YES
Q7: Were the outcomes measured in a valid and reliable way?	YES	YES	YES	YES
Q8: Was appropriate statistical analysis used?	YES	YES	YES	YES
Overall appraisal	INCLUDE	INCLUDE	INCLUDE	INCLUDE

**Table 6:** JBI Checklist for Cohort Studies.

Domain Questions (Q)	Chang Y 2021 [52]	Ji J 2022 [58]
Q1: Were the two groups similar and recruited from the same population?	YES	YES
Q2: Were the exposures measured similarly to assign people to both exposed and unexposed groups?	YES	YES
Q3: Was the exposure measured in a valid and reliable way?	YES	YES
Q4: Were confounding factors identified?	YES	YES
Q5: Were strategies to deal with confounding factors stated?	YES	YES
Q6: Were the groups/participants free of the outcome at the start of the study (or at the moment of exposure)?	YES	YES
Q7: Were the outcomes measured in a valid and reliable way?	YES	YES
Q8: Was the follow up time reported and sufficient to be long enough for outcomes to occur?	YES	YES
Q9: Was follow up complete, and if not, were the reasons to loss to follow up described and explored?	YES	YES
Q10: Were strategies to address incomplete follow up utilized?	N/A	N/A
Q11: Was appropriate statistical analysis used?	YES	YES
Overall appraisal	INCLUDE	INCLUDE

**Table 7:** JBI Checklist for Case-Control Studies.

Domain Questions (Q)	Lu HY 2021 [50]
Q1: Were the groups comparable other than the presence of disease in cases or the absence of disease in controls?	YES
Q2: Were cases and controls matched appropriately?	YES
Q3: Were the same criteria used for identification of cases and controls?	YES
Q4: Was exposure measured in a standard, valid and reliable way?	YES
Q5: Was exposure measured in the same way for cases and controls?	YES
Q6: Were confounding factors identified?	YES
Q7: Were strategies to deal with confounding factors stated?	YES
Q8: Were outcomes assessed in a standard, valid and reliable way for cases and controls?	YES
Q9: Was the exposure period of interest long enough to be meaningful?	YES
Q10: Was appropriate statistical analysis used?	YES
Overall appraisal	INCLUDE

**Table 8:** Synopsis of studies regarding lncRNAs in CKD.

Author/Year	Type of Study	Population and Sample Size	RNA	Mechanism/Axis	Result in Kidney Pathology	Diagnostic/Therapeutic
Okuyan HM 2021 [49]	clinical observational study	56 CKD patients and 20 healthy controls	lncRNA H19 upregulated	HMGB1/TLR4/NF-ĸB signalling pathway	In CKD patients negative relationship with glomerular filtration rate (GFR), positively correlated ferritin, phosphorus, parathyroid hormone, TNF-α, IL-6, OC, TAS and TOS levels.	clinical value in the pathogenesis of CKD and CKD-related complications
Lu HY 2021 [50]	translational research study	129 AKI patients and 100 healthy controls/HK-2 and HEK-293T cells	lncRNA MALAT1 was strongly elevated	lncRNA MALAT1 targets miR-204 through APOL1/NF-κB signaling.	AKI progression and inflammation	knockdown of lncRNA MALAT1/miR-204/APOL1/NF-κB axis may be a therapeutic target to the treatment of AKI.
Chang Y 2021 [52]	clinical observational study.	50 healthy controls, 100 CHF patients, 50 CHF + CKD patients	LncRNA-PVT1 downregulation	JNK/NF-κB and TNFα pathways	development of progressive CKD among patients with CHF.	PTV1 expression may have diagnostic and prognostic values for CKD.
Ma T 2022 [51]	translational research study that utilizes a multimodal experimental design	15 patients with AKI and 10 healthy controls. n = 6 per experimental group of mice subjected to the ischemia–reperfusion model. In vitro experiments with renal tubular epithelial cells, in a human cell line, and in mouse tubular epithelial cells.	lncRNA NEAT1_2, upregulated	NEAT1_2 upregulates the expression of FADD, CASP8, and CASP3 via inhibition of miR-129-5p	AKI-to-CKD transition by promoting TEC apoptosis	targeting NEAT1_2 expression may offer a therapeutic strategy to prevent TEC apoptosis and hinder the progression from AKI to CKD
Qiang Liu 2022 [48]	preclinical experimental research	60 rats (Sprague–Dawley)	lncRNA H19 upregulated	H19/miR-138/TLR3/NF-κB activation	Vascular calcification in CKD	management of high-phosphorus-mediated vascular calcification.
Xie LB 2020 [53]	preclinical experimental study.	Rats (Sprague–Dawley)/Human cells (HK-2)—at least 3 independent experiments,	LINC00963 was highly expressed	LINC00963 targets miR-128-3p to promote G1 arrest and apoptosis through JAK2/STAT1 pathway	Promotion of apoptosis, in hypoxia-induced HK-2 cells and I/R injury renal tissues	Blocking LINC00963 could target miR-128-3p to reduce the apoptosis and hinder the progression of AKI.
Puri B 2025 [46]	mechanistic laboratory study	NRK52E cells	lncRNA MALAT1 Upregulated	MALAT1/Smad2/3 pathway	AKI-to-CKD transition Apoptosis, fibrosis	targeting the MALAT1/Smad2/3 pathway could be a potential therapeutic target for mitigating fibrosis to prevent AKI-to-CKD transition.

**Table 9:** Synopsis of studies regarding miRNAs in CKD.

Author/ Year	Type of Study	Population and Sample Size	RNA	Mechanism/Axis	Result in Kidney Pathology	Diagnostic/Therapeutic
Xiaoyun G 2025 [54]	clinical observational study	126 patients with CKD, 126 healthy controls	miR-204 serum level lower in CKD	miR-204 impairs MUC4-dependent activation of the extracellular signal-regulated kinase signaling pathway	Reduction in miR-204 negatively correlated with renal function and positively correlated with eGFR, inflammatory factors increased by the reduction in miR-204	high diagnostic value for patients with chronic kidney disease
Newbury LJ 2021 [55]	translational research study	14 AKI patients, 10 healthy controls/6 mice (Sham group), 6 mice (IRI group)/cell lines (HK-2, mTEC)	Increase in miR-21, miR-126 and miR-141 and decreases in miR-192 and miR-204	protein tyrosine phosphatase receptor type G (PTPRG) as a direct miR-141 target -interaction with EGFR, miR-141/ZEB1/2- dependent pathway	Dysregulated EGFR, increased PTEC death and decreased cell viability, renal interstitial fibroblast activation	miR-21, miR-126, miR-141, miR-192 and miR-204 AKI biomarkers, miR-141 and miR-192 associated with AKI nonrecovery
Ahmed HM 2022 [57]	clinical observational study	60 pediatric patients with β-TM and 30 healthy children	β-TM and CKD patients had significantly lower miRNA-451	miR-451 (Ywhaz) product 14-3-3 zeta/repossession of nuclear factor FoxO3, prevention of Cat and GPx1 antioxidant enzymes	MiR-451 levels had significantly positive correlated with eGFR and reticulocyte counts	miRNA-451 has a protective role against CKD
Ji J 2022 [58]	clinical observational study.	142 children with sepsis-induced AKI	miR-320-3p upregulated in children with sepsis-induced AKI	370 target genes of miR-320-3p identified, including NRP1, SEMA3A and PTEN	Inhibition of NPR1 and SEMA3A exacerbate inflammatory response	prognosis prediction in children
Ni W 2024 [56]	preclinical experimental study	miR-26a-KO mice and wild type (WT) mice/HK2 and AC16 cells	miR-26a expression downregulated	Downregulation of miR-26a activated the LIMS1/integrin-linked kinase (ILK) signaling pathway	Inflammation and fibrosis	Supplementation with exogenous miR-26a attenuates heart and kidney injury in the context of CKD
Zhao JY 2020 [59]	preclinical experimental study	24 C57BL/6 male mice/HK-2 and 293T cells	MiR-101 is downregulated in AKI-CKD	COL10A1, COL12A1, and TGF-βR1 are the targets of miR-101	miR-101 is an anti-EMT; attenuates renal fibrosis; mitigates glomerular sclerosis, interstitial fibrosis, and inflammatory reaction	miR-101 may be a potential therapeutic miRNA for the treatment of renal fibrosis
Douvris A 2024 [60]	preclinical experimental study	114 Male Sprague Dawley rats	miR-486-5p (administration therapeutical)	down-regulation of proximal tubular activation of genes involved in apoptosis and tumor necrosis factor (TNF)-α signaling, down-regulation of PTEN expression	protects against ischemic AKI in rat, preserves early endothelial function, prevents peritubular capillary loss and tubulointerstitial fibrosis and preserves endothelium-dependent vasorelaxation	promising treatment against ischemic AKI

## Data Availability

The original contributions presented in this study are included in the article. Further inquiries can be directed to the corresponding author.

## Supplementary Materials

The following supporting information can be downloaded at: https://www.mdpi.com/article/10.3390/life16040579/s1, File S1: PRISMA 2020 Checklist. Reference [110] is cited in the supplementary materials.
